# Hearing Vocalizations during First Social Experience with Pups Increase Bdnf Transcription in Mouse Auditory Cortex

**DOI:** 10.1155/2023/5225952

**Published:** 2023-02-15

**Authors:** Amielle Moreno, Swetha Rajagopalan, Matthew J. Tucker, Parker Lunsford, Robert C. Liu

**Affiliations:** ^1^Neuroscience Graduate Program, Emory University, Atlanta, Georgia 30332, USA; ^2^Department of Biology, Emory University, Atlanta, Georgia 30322, USA; ^3^College of Science Undergraduate Neuroscience Program, Georgia Institute of Technology, Atlanta, Georgia 30332, USA; ^4^Center for Translational Social Neuroscience, Emory University, Atlanta, Georgia 30322, USA

## Abstract

While infant cues are often assumed to innately motivate maternal response, recent research highlights how the neural coding of infant cues is altered through maternal care. Infant vocalizations are important social signals for caregivers, and evidence from mice suggests that experience caring for mouse pups induces inhibitory plasticity in the auditory cortex (AC), though the molecular mediators for such AC plasticity during the initial pup experience are not well delineated. Here, we used the maternal mouse communication model to explore whether transcription in AC of a specific, inhibition-linked, memory-associated gene, brain-derived neurotrophic factor (*Bdnf*) changes due to the very first pup caring experience hearing vocalizations, while controlling for the systemic influence of the hormone estrogen. Ovariectomized and estradiol or blank-implanted virgin female mice hearing pup calls with pups present had significantly higher AC exon IV *Bdnf* mRNA compared to females without pups present, suggesting that the social context of vocalizations induces immediate molecular changes at the site of auditory cortical processing. E2 influenced the rate of maternal behavior but did not significantly affect *Bdnf* mRNA transcription in the AC. To our knowledge, this is the first time *Bdnf* has been associated with processing social vocalizations in the AC, and our results suggest that it is a potential molecular component responsible for enhancing future recognition of infant cues by contributing to AC plasticity.

## 1. Introduction

Across mammalian species, vocalizations play an important role in communicating vital information between conspecifics [1, 2]. Infants in particular use vocal signals to garner attention and convey needs to caregivers, whose parental response builds a mutual bond and ensures the infant's survival [3]. The role the auditory system plays in detecting and recognizing infant vocalizations [4] is thus evolutionarily critical.

Because of this crucial nature, mechanisms unlocking parental responses are often assumed to be innate, yet as caretakers gain experience with infants, plasticity at the genetic, molecular, and neuroendocrine levels does occur within the canonical regions mediating maternal behavior [5, 6]. Indeed, nature has allowed for so-called “open genetic programs” wherein experience is needed to refine how a behavior normally manifests [7]. In elucidating the mechanisms underlying refinement of parental behaviors though, even when models allow for changes within (usually subcortical) maternal circuits [8, 9], the sensorineural representations that trigger parental responses are not taken as plastic and are instead assumed already attuned to species-specific infant cues. While for some species this may hold, for certain sensory modalities where infant cues act as innate key stimuli [10], other modalities can still change through infant care experience in order to mediate adaptive parental responses. The auditory modality for pup-mother acoustic communication in mice is an instructive case, where increasing evidence supports a role for experience-dependent plasticity in how females respond adaptively to pup vocalizations.

Several studies have demonstrated that auditory cortical responses to pup ultrasonic vocalizations (USV), which normally elicit a search for and retrieval of displaced pups back to their nest [11], change after infant care experience in mice [12–14]. In particular, although traditional forms of experience-dependent cortical plasticity such as excitatory receptive field shifts and tonotopic map expansion do not occur [15], noncanonical changes tied to inhibitory mechanisms do. Specifically, neurons in regions of auditory cortex (AC) tuned outside of the frequency range of USVs become more suppressed by the sounds in mothers compared to pup-naïve virgins, potentially due to the action of parvalbumin-expressing interneurons, which play a key role in responding to pups with retrieval [16–19]. Functionally, auditory cortical plasticity apparently allows USVs to be better detected and discriminated [20], potentially helping to recognize pups from one's own litter [21]. Despite work [16, 18, 20, 22] confirming changes in sensory coding at the cortical (and even subcortical level [23]), the molecular mediators of this plasticity within auditory cortex are still being delineated. The neuropeptide oxytocin has been implicated in this plasticity [24–26], and estrogens have been hypothesized to prime the maternal auditory system to be plastic [27, 28]. However, little is known about plasticity-related genes within auditory cortex that may act at the time of initial social interaction with pups.

Here, we explored the hypothesis that the initial experience hearing vocalizations while interacting and providing pup care induces the transcription of brain-derived neurotrophic factor (*Bdnf*), which has long been known to mediate cortical plasticity and facilitate memory formation [29]. Mostly studied in other model systems, less is known about its activity in the auditory cortex [30–34]. The visual, somatosensory, and auditory cortices express BDNF during developmental critical periods and sensory stimulation to induce plasticity [35–38]. This memory-associated trophic factor is also affiliated with experience-dependent learning [39–41] and inhibitory plasticity [35], making it particularly relevant given the types of neurophysiological changes seen in AC during infant experience [16–19]. Moreover, the *Bdnf* mRNA isoform containing the 5′ exon IV displays activity-dependent transcription much like an immediate early gene [42], and this isoform is associated with sensory cortex expression [33, 43]. In addition, epigenetic modifications to *Bdnf*'s exon IV promoter region arise during learning and extinction processes in the cortex [44, 45], and such neuroepigenetic mechanisms for plasticity are increasingly being investigated within sensory cortex [46]. Finally, its gene contains an estrogen response element that could make it susceptible to hormonal modulation [47].

We investigated the molecular nature of AC plasticity by measuring *Bdnf* transcription evoked in the female mouse after the first social experience with pup USVs, controlling for estradiol exposure [48]. Subjects heard the playback of pup vocalizations, occurring either during pup retrieval (pup-experienced, Px) or outside of a social context (pup-naïve, Nv). We observed differential regulation of *Bdnf* exon IV specifically in the AC after pup calls were paired with maternal experience, though found no significant effect of systemic estrogen replacement. Our research suggests experiencing vocalizations associated with social behavior induces a transcriptional response in the AC, increasing the mRNA of this memory-associated gene. This finding highlights *Bdnf* as a potential player in socially induced sensory cortical plasticity.

## 2. Methods

### 2.1. Animal Use

The Emory University Institutional Animal Care and Use Committee approved all procedures involved in this study. Experiments were performed on adult virgin female CBA/CaJ mice with no prior adult experience caring for pups. Subjects were weaned at 21 days, placed in single-sex ALPHA-dri bedded housing with 2 to 5 per cage with a reverse-light cycle (14 hours of light/10 hours of dark) and access to food and water *ad libitum*. All experiments were performed during the animal's dark phase, under red light.

### 2.2. Hormonal Manipulations

**Figure 1:**
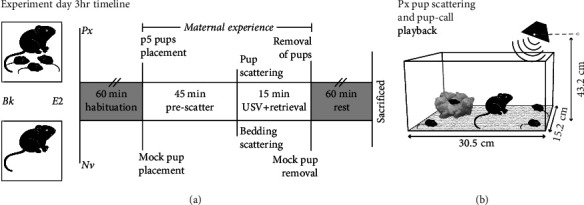
Experimental timeline. (a) Virgin female mice were ovariectomized and implanted with a blank (Bk) or estradiol (E2) implant. On the experiment day, half of the subjects received 1 hour (hr) of social experience with pups (Px), while the other half remained naïve (Nv) to pups. Experiments were run inside an anechoic chamber, beginning with 1 hr of solo habituation. Then, Px subjects experienced (top of timeline) 4 pups placed in their cage while Nv subjects experienced (bottom of timeline) mock pup placement. (b) After 45 min, pup-call stimuli were played for both conditions. For Px subjects, playback occurred during a pup scattering session; Nv subjects were subjected to a nest disturbance session to mimic pup scattering. Pups were removed (Px subjects) after 1 hr, and all subjects were sacrificed 2 hr after pup or mock pup placement, to assess gene transcription.

Animals (*N* = 104) were randomly divided into 1 of 2 hormonal implant treatments: estradiol (E2; *n* = 55) or blank (Bk; *n* = 51). Following previously published and validated protocols [23, 48], all mice were bilaterally ovariectomized and implanted with a subcutaneous capsule (2 mm Silastic tubing, sealed with silicone aquarium sealant) at the average age of 12 weeks (±11 days). Capsules contained either estradiol benzoate (E2) dissolved in sesame oil (50 *μ*l at 3 mg/ml, Selleck Chemicals LLC, Houston, TX, USA) or a blank (Bk) control containing only sesame oil (Figure 1(a)). The E2 concentration was previously found in our lab [23] to produce a plasma estradiol concentration comparable to levels reported in mouse mothers [49, 50]. They were sterilized using 0.9% saline solution and then hydrogen peroxide gas for 29 min. Subjects were singly housed and monitored for 3 days post surgery before experiment habituation (10 days post surgery ±4 days). The experimenters were blinded to implant type for behavior scoring.

### 2.3. Auditory Stimulus

We used two previously described sound stimuli [48, 51, 52]. Briefly, a 10 min long recording of concatenated pup calls was used on the experiment day as the pup-call playback stimulus. This stimulus contained a dense series of 1,055 pup-isolation calls grouped in bouts, ranging from 50 to 100 kHz, produced by 10 different CBA/CaJ mouse pups and averaging 55 dBSPL, with some calls reaching 95 dBSPL [48]. A second, 10 min long stimulus was created as a background noise stimulus by filtering out all calls from the pup-isolation call stimulus.

For a subset of 4 subjects, we measured actual emitted vocalizations from 4 donated pups placed in their cages. They produced an average of 389 calls in 45 min, nearly 3 times fewer calls than within our pup-isolation call stimulus. The sound pressure of the played-back recorded calls was comparable to what these live pups produced [53]. Hence, audio playback of the pup-isolation call stimulus ensured that all subjects were exposed to a minimum baseline of social sound during the pup scattering or mock pup scattering session on the experiment day.

### 2.4. Playback during Habituation and Experiment Day

After surgical recovery, subjects were further assigned to 1 of 2 different experimental groups: those remaining naïve to pups as adults (Nv) or those gaining 1 hr of pup experience (Px).

All subjects experienced habituation once a day for the 3 days prior to the experiment day to ensure familiarity with all nonpup stimuli associated with the experiment day. Habituation included procedure room transportation, portion-cup water access, anechoic chamber (44^″^ × 27^″^ × 24^″^, W × D × H inner dimensions, IAC Acoustics), exposure, and random playback of the background noise stimulus from a speaker at least 3 times per habituation. During playback, mock pup scattering sessions occurred involving a gloved experimenter gently distributing the subject's bedding material around their cage.

On the experiment day, female subjects were an average of 13 weeks of age (±12 days) and 10 days (±4 days) post ovariectomy surgery with implant placement. A portion of the subject's home cage bedding was removed and reserved for later use, and their cage was placed inside an anechoic chamber underneath a video camera. All subjects habituated solo inside the anechoic chamber for 1 hr.

For Px subjects, we removed pups from a donor dam's cage at postnatal day 5 or 6 (p5-6) and rubbed and situated them in the Px subject's reserved bedding. After the Px subject's solo habituation, video recording began and the nest with pups was transferred to a corner of the subject's cage. Px animals then experienced 45 min of uninterrupted social time with pups—their first exposure to pups as an adult. For Nv subjects, procedures were identical except that the reserved bedding without any pups was returned to the subject's cage.

After 45 min, we began the playback of the pup-call stimulus for both Px and Nv subjects, accompanied with pup scattering or mock pup scattering, respectively. Pup search and retrieval sessions involved multiple trials of scattering and retrieval. Each trial comprised removing 3 pups from the nest and scattering/placing them throughout the cage, while 1 pup was left in the established nest area (Figure 1(b)). Pup retrieval was characterized by the adult mouse approaching, picking up, and returning all displaced pups to the nest, completing a trial [54, 55]. Px animals were given ~4 min to complete a trial before the experimenter returned the pups to the nest. Thirty seconds after subject or experimenter retrieval, 3 pups were rescattered, initiating a new retrieval trial. Px subjects were classified as maternally responsive “retrievers” if they successfully retrieved all 3 pups on 3 or more trials. To ensure adequate pairing between pup calls and maternal behavior response, scatterings continued through the 10 min playback and into a subsequent 5 min period, as needed to meet retriever status. Nv subjects had small portions of their bedding material scattered at least 5 times during their mock pup scattering session.

After a total of 1 hr of pup experience, we removed the donated pups from the cage of the Px subjects and simulated the same disturbance for Nv subjects. The subjects then remained in the anechoic chamber for another 1 hr before being sacrificed to assess gene transcription.

### 2.5. Behavior Assessment

Video of the subject during 1 hr of pup experience was monitored online for aggressive behavior and recorded onto DVD for later common maternal behavioral scoring using the Observer XT application (Noldus Information Technology, Leesburg, VA, USA). All video scoring was performed blind to the hormonal condition of the subject. Behaviors were judged as mutually exclusive from one another. Common maternal behaviors including licking/grooming, nest building, crouching over a nest with pups, and the latency to retrieve a displaced pup were scored for duration and number of instances, according to established ethogram procedures [56].

Repetitive head bobbing of the adult in close proximity of a pup in the nest, leading to the gentle rocking of the pup, was judged as sniffing/licking/grooming.

We inferred crouching from an adult mouse's stationary position over the nest with her ventral side bowed out towards the pups [54, 57]. Hovering consists of the adult mouse laying or sitting on top of the nest [55]. Because of difficulty distinguishing these 2 behaviors on video, they were scored as the same. We distinguished this behavior by an increase in the animal's apparent size due to sedentary position and minimal head movement. Walking over the nest was not scored as hovering/crouching.

Nest building was scored when the adult mouse moved or displaced the bedding material around the established nest or pups or otherwise manipulated it. This did not include walking over the nest, because any movement of the bedding was likely inadvertent [54].

These three behaviors—licking/grooming, crouching, and nest building—were scored if they persisted for more than 2 seconds [54, 55, 57, 58]. Totals were calculated for the first 45 min (prescattering) and over the 1 hr of pup experience, which included the 15 min pup retrieval session (Figure 1(b)).

Mice can display rapid onset of retrieval behavior while other rodents, such as rats, may take days, if inexperienced with infant care [12]. During a retrieval trial, the latency to retrieve began when the experimenter removed 3 pups from the nest and ended the moment the adult mouse let go of the final displaced pup in the nest. Nest delivery was necessary to count as a retrieval [58]. Three pups scattered mean that three retrievals were possible per trial. We also recorded the time to retrieve the 1^st^, 2^nd^, and 3^rd^ pups during each retrieval trial.

Both ovariectomized and intact nulliparous female mice are known to engage in maternal behaviors when exposed to pups [59], although aggression towards pups does occur [60]. Attack behavior was grounds for the immediate removal of pups and end of experiment. Of the 104 animals in this study, 72 were Px females, of which 21 attacked their foster pups (Bk = 11 and E2 = 10), usually within 10 min of placement. There was no difference in rate of attack between the 2 hormonal conditions (Fisher's exact test: *p* = 0.612). Animals that attacked were not included in behavior scoring or brain processing.

Total nonaggressive animals formed the following groups: PxE2 (*n* = 27), PxBk (*n* = 24), NvE2 (*n* = 18), and NvBk (*n* = 14). Of these, we scored behavior from 69 animals; 14 others could not be scored due to technical recording issues. Two PxE2 animals were removed from the analyses because line of sight was obscured. One PxE2 animal was removed from the total 1 hr, but not the first 45 min analysis, because a technical failure caused no auditory stimulus playback. Of the 69 subject videos scored, 3 failed to retrieve scattered pups (2 PxE2 and 1 PxBk) and an additional 2 animals did not display maternal behavior including retrieval (1 PxE2 and 1 PxBk).

### 2.6. Tissue Collection and Processing

One hour after pup or mock pup experience was concluded, subjects were incapacitated with CO_2_ and decapitated. Brains were removed via fresh frozen extraction using liquid nitrogen and Tissue-Tek OCT compound (Sakura Fine Technical, Tokyo, Japan) and stored at -80°C. The mouse brain atlas was referenced to visually approximate brain level during microtome sectioning and tissue sample collection [61]. Starting at the first rostral presentations according to atlas figure positions during rostral to caudal sectioning, tissue from the medial preoptic area (MPOA), the primary auditory cortex (AC), the primary visual cortex (V1), and the ventral hippocampus (VH) was collected bilaterally via a 1 mm microtissue puncher. To avoid collection of adjacent areas, we conservatively collected between atlas figure sections 34-39 for the MPOA (~0.78 mm), 50-60 for the AC (~1.22 mm), 51-55 for the V1 (~0.62 mm), and 55-61 for the VH (~0.72 mm) adjusting for width. Tissue was stored in 70 microliters (*μ*l) of RNAlater (Thermo Fisher Scientific, Waltham, MA, USA) at -20 C° until RNA isolation.

Total RNA was extracted from tissue using the RNeasy mini kit (Qiagen, Hilden, Germany) according to the manufacturer's protocol. On-column DNAse (Qiagen) treatment eliminated genomic DNA. Purity of RNA was assessed by 260/280 ratio and concentration analyzed using Gen5 Take3 software on a BioTek Synergy HT RNA quantification machine (BioTek, Winooski, VT, USA). RNA was reverse transcribed into cDNA using the superscript III reverse transcriptase kit (Invitrogen, Thermo Fisher Scientific, Waltham, MA, USA), standardized to 150 ng/20 *μ*l using RNA concentration measurements.

Quantitative real-time polymerase chain reaction (qRT-PCR) was conducted with validated TaqMan® probes and TaqMan® Universal PCR Master Mix (Applied Biosystems, Austin, TX, USA) on the ABI Step-One-Plus PCR system using Step-One software by Life Technologies (Applied Biosystems). Each sample well contained the following: 10 *μ*l of PCR Master Mix, 1.7 *μ*l of cDNA (150 ng/20 *μ*l), 7.3 *μ*l of nuclease-free water (Sigma-Aldrich, St Louis, MO, USA), and 1 *μ*l of the specific TaqMan® Gene Expression Assay. Each qRT-PCR run contained the following: 5 samples for each of the 4 experimental conditions, in duplicate for the gene of interest and a positive reference gene; a no-RT sample; and a water sample for each primer as internal controls. The following schedule was used for the qRT-PCR program: 50°C for 2 min and 95°C for 10 min and cycling stage: 95°C for 15 sec and then 60°C for 1 min for 40 cycles. Each qRT-PCR result was verified with an additional run of independent samples (see Statistical Analysis in Methods).

Experimental TaqMan® primers for mice (Thermo Fisher Scientific) included one for the 3′ coding exon of *Bdnf* exon IX (Mm04230607_s1; coding to Chr.2: 109,674,700–109,727,043) which allows for the evaluation of total *Bdnf* transcription (i.e., the sum of all isoforms transcribed). Another primer identified the site-specific transcription of the *Bdnf* exon IV isoform (Mm00432069_m1; coding to Chr2: 109,692,436-109,692,774). Housekeeping primers included *β*-actin (Mm02619580_g1 Chr.5: 142,903,116–142,906,724, used for MPOA, AC, and V1) and ribosomal protein eL19 (RPL19; Mm02601633_g1, Chr.11: 98,023,080–98,030,493, used for VH; Thermo Fisher Scientific).

Of the 82 Nv and nonattacking Px animals that experienced our paradigm, 68 were used for qRT-PCR analyses. The samples from 12 animals were consumed during processing and protocol optimization and did not contribute to *Bdnf* mRNA data.

### 2.7. Statistical Analysis

Data were analyzed with JMP data analysis software (SAS, Cary, NC, USA). Behavioral data were screened for outliers, and points that met the JMP software's Robust Fit (Huber's M-estimation method) criteria were removed [62]. Maternal behavioral data were assessed during both the first 45 min and for the total 1 hr, which included real or mock pup scattering and pup-call playback.

To determine an appropriate sample size, we conducted a power analysis (G^∗^Power software, RRID:SCR_013726, Dusseldorf, Germany) by performing a literature search for previous studies examining sensory cortical plasticity as measured by *Bdnf* mRNA in adult female mice. We consulted 4 previous neuroendocrinology studies in relation to *Bdnf* transcription or protein levels [32, 63–65]; two of these also covered sensory cortical changes. Due to the required number of controls and sample duplicates, as well as the constraints of qPCR hardware, we believed a sample size of *N* = 20 would be adequate to detect transcriptional changes associated with auditory experience.

Fisher's exact test determined the proportions of attacking subjects per hormone condition. Other data were subjected to statistical analyses using two-way ANOVA for experiments with 4 groups and Student's *T*-test for comparing 2 groups. Prior to analysis, experimental groups were checked for unequal variances using the Levene test. A significant Levene test prompted the use of the nonparametric Wilcox/Kruskal-Wallis test over the Student's *T*-test for two group comparisons and Spearman's correlation coefficient over the pairwise correlations analysis for multivariate analysis. All hormone analyses were conducted under the hypothesis that E2 animals would be more likely to express maternal behavior [28, 66, 67] prompting one-tailed Student's *T*-tests. We also held the hypothesis that Px subjects would have lower cycles to threshold (Ct's) than Nv subjects for *Bdnf* transcripts in our qRT-PCR, indicating more transcription of *Bdnf* mRNA in response to the social experience gained in our paradigm [40, 45]. Differences were considered to be statistically significant at *p* < 0.05 and expressed as mean ± SEM.

Data from qRT-PCR were analyzed using the 2-*ΔΔ*Ct method [68]. Data were normalized to the naïve blank (NvBk) condition for two-way ANOVA and Nv for Student's *T*-test. The average ΔCt value data were used for analyses and were the result of average Ct for a sample's gene of interest (all replicates) minus the average Ct for that same sample's reference control gene. Reference control genes were verified using the 2^-*Δ*Ct^ method, where ΔCt is the average of a sample's replicate Ct minus the average Ct of the normalized group [69]:

(1) $$\begin{array}{c} 2^{-\left[\text{Ct }\left(\text{average of each sample's replicates}\right)-\text{Ct }\left(\text{average of normalized group's replicates}\right)\right]}. \end{array}$$

**Figure 2:**
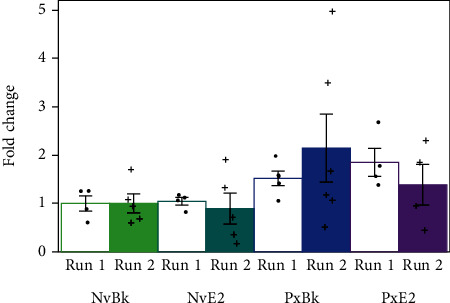
All data for Bdnf exon IV transcription in AC from two runs with independent samples. Fold change data from both independent qRT-PCR runs of AC samples from the 4 animal groups where pup experience and hormones were manipulated. Error bars here and in subsequent figures represent mean ± standard error.

Multiple brain regions were tested for their transcription of *Bdnf* mRNA all exons or exon IV, to determine an effect of hormones and experience using a 2 × 2 ANOVA. Each qRT-PCR for a brain region and *Bdnf* transcript type was conducted twice, with different 20 subjects for each run, to ensure that the reported results were reproducible. The results of the most conservative runs (largest ANOVA *p* values) are reported in Results, but for completeness, the underlying data (Figure 2) and statistical outcomes (Table 1) from both independent runs that support a significant reported result are presented here.

The MPOA was initially a region of interest because it had previously demonstrated significant increase in the immediate early gene c-Fos in both intact and ovariectomized nulliparous female mice with 30 min of pup experience [60]. However, we found a significant effect of hormones on the expression of our endogenous control gene, *β*-actin, in the MPOA during verification (*F*_3,17_ = 8.2627, *p* = 0.011) and therefore could not verify *Bdnf* expression for the MPOA. Further research is needed to determine whether *β*-actin transcription in the MPOA changes with hormone treatment. All PCR data reported showed no significant effect of hormone or experience on housekeeping gene's average ΔCt.

## 3. Results

### 3.1. Systemic Estrogen Modulated Overall Maternal Behavior but Not Pup Retrieval in Px Females

We first confirmed that the social context with pups measurably affected the virgin Px and Nv subjects by analyzing nest building over the full 1 hr period including sound playback, since this was the only behavior among the typical maternal repertoire [70] that both Px and Nv could perform whether there were pups present or not. With pups present, Px mice spent significantly more time than Nv engaged in nest building (two-way ANOVA, *F*_(3, 62)_ = 23.8919, *p* < 0.0001; not shown), as expected. There was neither a main effect of hormone exposure (two-way ANOVA, *F* = 0.4466, *p* = 0.5064) nor an interaction between hormone and experience variables (two-way ANOVA, *F* = 0.455, *p* = 0.5025) on nest building.

**Figure 3:**
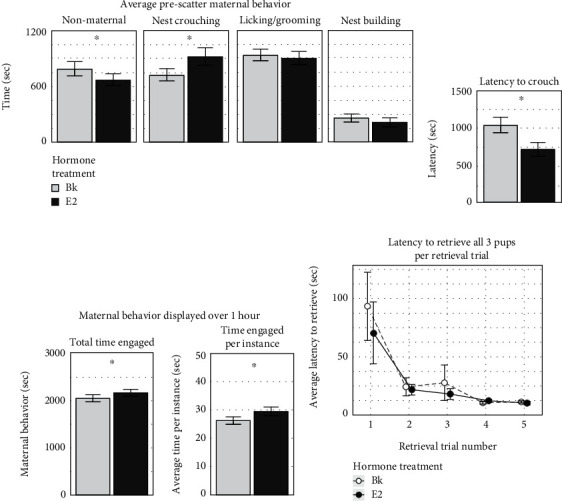
E2 affected the amount of maternal behavior in seconds (sec) displayed by pup-exposed subjects (Px) over the course of 1 hr of pup experience but did not affect pup retrieval behavior. (a) During the first 45 min of pup experience, the PxBk group showed more time engaged in nonmaternal behaviors than the PxE2 group (*p* = 0.02). The PxE2 group spent more time engaged in nest crouching behavior (*p* = 0.04). Time engaged in licking/grooming (*p* = 0.62) and nest building (*p* = 0.69) was not significantly different between hormone treatment. (b) PxE2 subjects had a shorter latency before crouching over a nest of foster pups compared to PxBks (*p* < 0.01). (c) During the total 1 hr of pup experience, PxE2s spent significantly more time engaged in maternal behaviors than PxBk subjects (*p* = 0.04, *n* = 42) and more time per instance (*p* = 0.04). (d) During pup call and retrieval pairing, the E2 subject's latency to retrieve all 3 pups was not significantly lower than Bk subjects, including the first retrieval trial (*p* = 0.81). ^∗^ indicates *p* values < 0.05. Error bars represent SEM.

We next checked whether our E2 manipulation affected the maternal behavior of Px animals, since given E2's intrinsic association with maternal behavior [66, 71, 72], we expected that E2 would increase the time Px subjects engaged with pups, thereby enhancing social interactions. Indeed, during the 45 min prescatter period when experimenter-initiated pup scattering did not artificially drive a competition between pup retrieval and other behaviors, PxBk subjects spent significantly more time engaged in nonmaternal behaviors (one-tailed Student's *T*-test: *p* = 0.02; Figure 3(a)). In terms of specific maternal behaviors, PxE2 subjects had significantly lower latency to crouch over pups (one-tailed Student's *T*-test: *p* < 0.01), which likely contributed to more time crouching than PxBk subjects (one-tailed Student's *T*-test: *p* = 0.04; Figures 3(a) and 3(b)). There was no difference in the prescatter time licking/grooming (one-tailed Student's *T*-test: *p* = 0.62) or nest building (one-tailed Student's *T*-test: *p* = 0.69; Figure 3(a)) between the PxE2 and PxBk animals.

Over the full 1 hr period, PxE2 subjects spent significantly more total time displaying maternal behaviors generally than PxBk animals (one-tail Student's *T*-test: *p* = 0.04; Figure 3(c)). PxE2 animals also spent more time engaged in maternal behavior per instance (one-tail Student's *T*-test: *p* = 0.04; Figure 3(c)). Hence, our hormone manipulation had a significant effect on the time subjects engaged in maternal behavior, providing a positive control for the behavioral effects of our E2 implants and enhancing social interactions.

This enhancement, however, did not translate into measurable differences in pup retrieval during the 15 min pup scattering and pup-call playback session. The likelihood of successfully retrieving all 3 pups during the first retrieval was no greater for PxE2- than PxBk-treated subjects (Fisher's exact test: *p* = 0.47). The latency to successfully retrieve their first pup during the first scattering was not significantly different between hormone groups (two-tailed Student's *T*-test: *p* = 0.22). Also, the latency to retrieve all pups during the first scattering was not lower for PxE2 subjects (two-tailed Student's *T*-test: *p* = 0.81, Figure 3(d)). There was no difference in the average number of pups either group retrieved (two-tailed Student's T-test: *p* = 0.49). Regardless of hormone treatment, Px subjects took the longest time on their first retrieval trial to retrieve all 3 pups (one-way ANOVA, *F*_(3,114)_ = 21.126, *p* < 0.0001). Thus, even though increased time spent engaged in other maternal behaviors indicated that the systemic E2 implant had a behavioral effect in Px mice, hormone treatment neither enhanced nor interfered with retrieval behavior during the pup scattering session.

### 3.2. Bdnf Exon IV Transcription in AC Increased after the First Social Sound Experience with Pups

Our primary interest was to test for increased transcription of a plasticity-related gene due to the first experience hearing social sounds in an ethological auditory behavior. We measured total *Bdnf* mRNA transcripts, and the exon IV-specific transcript, from three brain regions (AC, V1, and VH) of virgin female mice shortly after their first pup experience. The exon IV isoform was chosen given its association with activity-dependent induction [42], memory formation [40], and the epigenetic regulation of adjacent histones mediated by E2 and learning tasks [32].

We conducted two independent runs using different animals for each qRT-PCR for the two *Bdnf* isoforms and three brain areas, under certain assumptions about the number of animals needed per run (see Methods). Reporting the run with the most conservative outcome, a two-way ANOVA with pup experience and hormone treatment as factors yielded no significant main effects or interactions (Table 2). The results were consistent across both runs for V1, which was our negative control brain area since the experiment took place during subjects' dark phase under red filtered light, and for VH, which has been implicated in social engrams [73]. However, for the main site of interest in auditory plasticity, the AC, inconsistent outcomes between the two runs for exon IV (Table 1), led us to consider a post hoc analysis in case we may have been underpowered to detect effects of both experience and hormones on *Bdnf* transcription. Since the behavioral analysis revealed that hormone treatment did not impact pup retrieval during sound playback—the period when a social function of pup calls could be learned—we focused on the role of experience.

**Figure 4:**
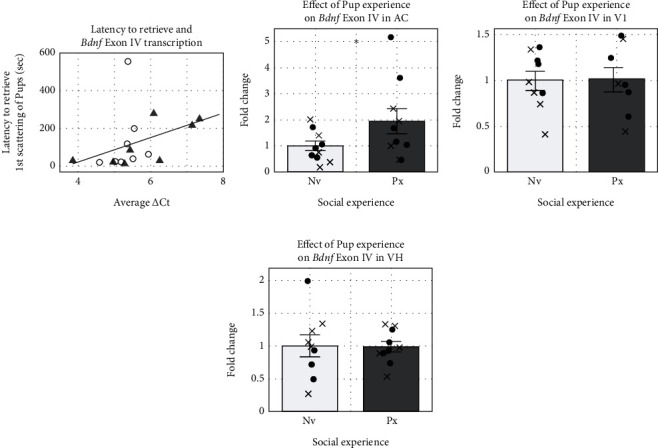
Pup experience while hearing pup calls increased Bdnf exon IV transcription specifically in AC. (a) In AC, more Bdnf exon IV transcripts was significantly associated with shorter latency to retrieve pups in the first scattering trial. Data from runs 1 (triangles) and 2 (open circles) combined. (b) Pup experience significantly increased Bdnf exon IV transcription in AC. Data from Bk (filled circles) and E2 (x) pooled for both Nv and Px subjects. (c) In V1, the mRNA from Bdnf exon IV demonstrated no significant difference between conditions. (d) In VH, the mRNA from Bdnf exon IV demonstrated no significant difference between conditions. ^∗^ indicates *p* value < 0.05.

We combined available behavioral and ΔCt data from runs 1 and 2, collapsing across Bk and E2 animals, to gain insight into whether retrieval experience may be correlated with AC *Bdnf* exon IV transcription (Table 1). Indeed, shorter latencies to retrieve pups were significantly associated with lower ΔCt indicating more *Bdnf* transcripts (Figure 4(a)). This motivated testing solely for the effect of experience in each run, where we found a consistent significant effect (Table 1 and Figure 2), reported here for the most conservative outcome (Figure 4(b)). As additional controls for specificity, no significant effect of experience was seen for the negative control site V1 (one-tailed Student's *T*-test: *p* = 0.506; Table 2 and Figure 4(c)) or the nonauditory social memory site VH (one-tailed Student's *T*-test: *p* = 0.338; Table 2 and Figure 4(d)).

## 4. Discussion

Our study provides the first evidence that *Bdnf* transcription increases in the AC after social auditory experience in an exon transcript-specific manner. *Bdnf*'s exon IV mRNA concentrations nearly doubled in the mouse AC when pup calls and pup experience were paired compared to when the vocalizations alone were heard outside of a social context. This result adds a molecular indicator that AC physiological changes after hearing infant calls are likely due to experience-dependent plasticity rather than a consequence of maternal hormone-mediated latent states [74]. In fact, systemic E2 treatment did not significantly influence the transcription of total or exon IV *Bdnf* mRNA within the AC, V1, or VH, whether pups were present or not during pup-call playback.

### 4.1. Experience-Dependent Auditory Cortical Plasticity Induced by BDNF

Maternal experience fosters familiarity with a new set of infant-emitted vocalizations. While we have previously demonstrated how the molecular response in AC to vocalizations differs between individuals that find the sounds familiar or not [48, 52], here we wanted to identify molecular events that help initiate AC plasticity at the *first* instance when vocalizations become associated with pups. One main form of AC plasticity to emerge after pup experience is altered inhibition [16, 18, 19, 75]. USVs elicit a stronger call-inhibited response in mothers compared to virgin females within so-called lateral band regions of core auditory cortex [16]. In fact, parvalbumin-expressing inhibitory interneurons in mothers shift their best frequency up an octave and closer to USV frequencies, relative to virgins [76]. These changes are thought to improve neural population contrast in the cortical representation of pup vocalizations by inhibiting activity in regions that should not be responding to the USVs [17, 27].

Given the apparent importance of altering evoked inhibition, we focused on a key molecule associated with inhibitory signaling and plasticity within the cortex, BDNF [77–79]. BDNF is released by pyramidal cells, targeting GABAergic interneurons and, in turn, regulating excitation in the neocortex and hippocampus [39]. Parvalbumin-positive interneurons abundantly express BDNF's TrkB receptors [80], and under this neurotrophic factor's influence, these interneurons increase their production of GABA and its synthesizing enzyme [81, 82]. The *Bdnf* exon IV transcript is regulated by neuronal activity in an immediate early gene-like fashion [42, 43] [83, 84] and is associated with memory formation [40]. Hence, the acute pup experience-dependent increase in *Bdnf* exon IV mRNA we found implies that the social context of the auditory experience is particularly potent in engaging this inhibitory plasticity-related molecular pathway, which may give rise to the stronger evoked inhibition seen in physiology studies [16, 17], potentially helping to imbue the sounds with social meaning.

### 4.2. Effect of Estrogens on Auditory Processing

We did not observe a direct effect of systemic estrogen replacement on *Bdnf* transcription in AC, despite a positive, expected influence of our E2 implant on total time engaged in maternal behavior, especially nest crouching [85]. Nevertheless, other studies have implicated estrogens in processing ethologically relevant sensory stimuli in songbirds [86] and a variety of other vertebrate species [87]. In fact, we have previously hypothesized that rising estrogen levels during pregnancy may prime the maternal brain and auditory system to rapidly respond to pup-associated cues and maintain this plasticity [17, 27, 88], and we found that estrogen replacement differentially impacts subcortical activity in response to pup calls [48]. At the level of the mammalian sensory cortex, Clemens et al. [89] discovered that natural cycling estrogens influenced excitability of fast-spiking interneurons in whisker barrel cortex of female rats in response to a conspecific's social touch. Hence, the lack of an estrogen effect in the current study may be more indicative of limitations in our approach rather than a definitive absence of any estrogenic modulation of AC plasticity.

For example, our manipulation of estrogens was systemic, based on ovariectomizing and replacing gonadal estrogens with a Silastic implant—a commonly used method for controlling hormone exposure [23, 48]. However, implants cannot mimic the normal physiological fluctuations in hormone concentrations in the brain. In fact, there is increasing evidence that brain-derived neuroestrogens rather than gonadal estrogens can play an important role in neuromodulation. The song-bird auditory forebrain circuit experiences rapid regulation by neuroestrogens during sound processing in social contexts [86, 90, 91]. In rodents, the presence of aromatase in axonal processes also suggests rapid neuroestrogen modulation of neural activity via activation of kinase cascades [92, 93]. A publication utilized a forebrain neuron-specific aromatase knockout (FBN-ARO-KO) mouse to block all synthesis of neuroestrogens in forebrain excitatory neurons [94]. Ovariectomized FBN-ARO-KOs experienced deficits in forebrain spine and synaptic density as well as spatial, recognition, and contextual fear memory. However, heterozygous, ovariectomized females demonstrated no change in BDNF, pERK, or pCREB levels in the cortex, even though these memory-associated markers decreased in ovariectomized FBN-ARO-KOs. In light of this finding, our negative E2 *Bdnf* results (despite positive behavioral effects on maternal behavior) might reflect compensation for the loss of gonadal E2 in ovariectomized Bk animals by an upregulation of local brain-derived neuroestrogen modulation.

## 5. Conclusion

We demonstrated that hearing natural pup vocalizations during pup experience enacted immediate transcriptional changes in the AC. Specifically, the *Bdnf* exon IV transcript, which is associated with neuronal activity and experience-dependent synaptic plasticity, increases in the AC when female mice experience infant vocalizations in a social compared to nonsocial context. The transcript type suggests a link to inhibitory neuron plasticity, which has been observed in mouse AC as a consequence of motherhood and infant experience. This increase in transcription during the initial pup experience is potentially one of the first transcriptional changes in a series, leading to synaptic and then circuit level modifications improving a caretaker's AC representation of vocalizations. Thus, our findings add to previous AC research indicating experience caring for infants enacts molecular changes and lasting enhanced processing of infant vocalizations.

## Figures and Tables

**Table 1:** Statistical analyses for Bdnf exon IV transcription in AC from two runs with independent samples.

Statistics		*AC Bdnf* exon IV transcript (average ΔCt)	
		Run 1	Run 2
2 × 2 ANOVA	*Nv*	Bk 6.4 ± 0.4; E2 5.9 ± 0.1	Bk 6.2 ± 0.2; E2 6.8 ± 0.6
	*Px*	Bk 5.4 ± 0.1; E2 5.1 ± 0.2	Bk 5.4 ± 0.5; E2 5.9 ± 0.5
	*ANOVA*	*F*_3,14_ = 3.889, *p* = 0.034^∗^	*F*_3,17_ = 1.368, *p* = 0.288
	Hormone	*F* = 1.888, *p* = 0.192	*F* = 1.04, *p* = 0.323
	Experience	*F* = 9.508, *p* = 0.008^∗^	*F* = 2.66, *p* = 0.121
	Hormone x experience	*F* = 0.080, *p* = 0.781	*F* = 0.002, *p* = 0.961

Spearman's *ρ*: correlation with *Bdnf*	Latency to retrieve	*r* (14) = 0.675, *p* = 0.004^∗^	

Student's *T*-test for experience	One-tailed Px > Nv	*p* = 0.003^∗^	*p* = 0.044^∗^

**Table 2:** Results for Bdnf All and exon IV transcription in AC, V1, and VH from the most conservative runs.

Brain region	Statistics	*Bdnf* transcript (average ΔCt)	
		All	Exon IV
AC	*Nv*	Bk 5.6 ± 0.7; E2 6.2 ± 0.5	Bk 6.2 ± 0.2; E2 6.8 ± 0.6
	*Px*	Bk 5.3 ± 0.2; E2 5.1 ± 0.1	Bk 5.4 ± 0.5; E2 5.9 ± 0.5
	*ANOVA*	*F*_3,14_ = 0.957, *p* = 0.442	*F*_3,17_ = 1.368, *p* = 0.288
	Hormone	*F* = 0.232, *p* = 0.637	*F* = 1.040, *p* = 0.323
	Experience	*F* = 1.902, *p* = 0.191	*F* = 2.669, *p* = 0.121
	Interaction	*F* = 0.670, *p* = 0.427	*F* = 0.002, *p* = 0.961
	Student's *T*-test for experience	Nv mRNA < Px mRNA *p* = 0.087	Nv mRNA < Px mRNA *p* = 0.044^∗^

V1	*Nv*	Bk 5.1 ± 0.2; E2 4.4 ± 0.5	Bk 0.2 ± 0.1; E2 0.8 ± 0.2
	*Px*	Bk 5.1 ± 0.3; E2 5.0 ± 0.3	Bk 0.4 ± 0.2; E2 0.7 ± 0.5
	*ANOVA*	*F*_3,15_ = 0.820, *p* = 0.504	*F*_3,14_ = 0.726, *p* = 0.554
	Hormone	*F* = 1.221, *p* = 0.287	*F* = 1.811, *p* = 0.201
	Experience	*F* = 0.539, *p* = 0.474	*F* = 0.051, *p* = 0.824
	Interaction	*F* = 0.563, *p* = 0.465	*F* = 0.239, *p* = 0.632
	Student's *T-*test for experience	Nv mRNA < Px mRNA *p* = 0.79	Nv mRNA < Px mRNA *p* = 0.506

VH	*Nv*	Bk 2.4 ± 0.3; E2 2.4 ± 0.4	Bk 7.5 ± 0.4; E2 7.6 ± 0.4
	*Px*	Bk 2.1 ± 0.2; E2 3.1 ± 0.4	Bk 7.4 ± 0.1; E2 7.4 ± 0.2
	*ANOVA*	*F*_3,15_ = 1.249, *p* = 0.329	*F*_3,16_ = 0.06, *p* = 0.98
	Hormone	*F* = 1.832, *p* = 0.197	*F* = 0.012, *p* = 0.914
	Experience	*F* = 0.131, *p* = 0.722	*F* = 0.148, *p* = 0.705
	Interaction	*F* = 1.656, *p* = 0.219	*F* = 0.011, *p* = 0.916
	Student's *T-*test for experience	Nv mRNA < Px mRNA *p* = 0.683	Nv mRNA < Px mRNA *p* = 0.338

## Data Availability

The behavioral and molecular data used to support the findings of this study are available from the corresponding author upon request.

## Additional Points

*Highlights*. (i) AC Bdnf exon IV increased only when vocalizations occur in a social context. (ii) E2 affected crouching and time engaged with pups but not retrieval. (iii) E2 did not affect AC Bdnf mRNA's response to social auditory cue experience. (iv) Bdnf mRNA did not increase in ventral hippocampus to social auditory cue.
